# Metal Decoration Effects on the Gas-Sensing Properties of 2D Hybrid-Structures on Flexible Substrates

**DOI:** 10.3390/s151024903

**Published:** 2015-09-25

**Authors:** Byungjin Cho, Jongwon Yoon, Sung Kwan Lim, Ah Ra Kim, Sun-Young Choi, Dong-Ho Kim, Kyu Hwan Lee, Byoung Hun Lee, Heung Cho Ko, Myung Gwan Hahm

**Affiliations:** 1 Advanced Functional Thin Films Department, Surface Technology Division, Korea Institute of Materials Science (KIMS), 797 Changwondaero, Sungsan-Gu, Changwon, Gyeongnam 642-831, Korea; E-Mails: kimahra86@kims.re.kr (A.R.K.); tjsdud2995@kims.re.kr (S.-Y.C.); dhkim2@kims.re.kr (D.-H.K.); 2 School of Materials Science and Engineering, Gwangju Institute of Science and Technology (GIST), 261 Cheomdan-gwagiro, Buk-Gu, Gwangju 500-712, Korea; E-Mails: jwyoon@gist.ac.kr (J.Y.); bhl@gist.ac.kr (B.H.L.); heungcho@gist.ac.kr (H.C.K.); 3 Department of Nanobio Materials and Electronics, Gwangju Institute of Science and Technology (GIST), 261 Cheomdan-gwagiro, Buk-Gu, Gwangju 500-712, Korea; E-Mail: lsk8410@gist.ac.kr; 4 Electrochemistry Department, Surface Technology Division, Korea Institute of Materials Science (KIMS), 797 Changwondaero, Sungsan-Gu, Changwon, Gyeongnam 642-831, Korea; E-Mail: lgh1636@kims.re.kr

**Keywords:** MoS_2_, graphene, 2D hybrid-structure, metal decoration, flexible gas sensor

## Abstract

We have investigated the effects of metal decoration on the gas-sensing properties of a device with two-dimensional (2D) molybdenum disulfide (MoS_2_) flake channels and graphene electrodes. The 2D hybrid-structure device sensitively detected NO_2_ gas molecules (>1.2 ppm) as well as NH_3_ (>10 ppm). Metal nanoparticles (NPs) could tune the electronic properties of the 2D graphene/MoS_2_ device, increasing sensitivity to a specific gas molecule. For instance, palladium NPs accumulate hole carriers of graphene/MoS_2_, electronically sensitizing NH_3_ gas molecules. Contrarily, aluminum NPs deplete hole carriers, enhancing NO_2_ sensitivity. The synergistic combination of metal NPs and 2D hybrid layers could be also applied to a flexible gas sensor. There was no serious degradation in the sensing performance of metal-decorated MoS_2_ flexible devices before/after 5000 bending cycles. Thus, highly sensitive and endurable gas sensor could be achieved through the metal-decorated 2D hybrid-structure, offering a useful route to wearable electronic sensing platforms.

## 1. Introduction

Two-dimensional (2D) graphene has evoked considerable interest in diverse potential applications [1,2,3,4,5]. 2D graphene has been developed for a long time and primarily employed as a transparent conductor for electronic applications [6] and indeed, it is in the middle of vital advances toward practical applications [7]. In recent years, 2D layered transition metal dichalcogenides (TMDs) have been also attracting much attention since they possess extremely large surface-to-volume ratios, low power consumption, high process compatibility, and inherent flexibility [8,9,10,11,12,13,14,15,16]. The successful demonstration of 2D MoS_2_ for based field-effect transistors [17,18,19], photodetectors [20,21], and gas sensors [22,23,24,25,26] have proved its outstanding physical properties.

The ultimate goal of the 2D nanomaterial research field is to atomically build 2D architectures with various van der Waals (vdW) interactions, which is referred to as the vdW heterostructure [27,28,29]. Strong covalent bonds in-plane and relatively weak vdW interactions out-of-plane provide the structural stability for these atomic-scale heterostructures [27]. Thus, various types of the heterostructures have been intensively investigated recently [28,29]. In particular, intense studies have been concentrated on 2D TMD-graphene hybrid-system for field-effect transistor [30] and photonic applications [31]. There are few studies about sensing platforms consisting of all 2D materials, which must be one of breakthrough pathways toward next-generation electronic sensors. Also, functionalization on the 2D materials is essentially required for tuning and optimizing gas-sensing performance. Furthermore, reliable gas-sensing capability of the 2D materials for a futuristic wearable sensing component should be established on a flexible substrate. In this context, gas-sensing tunability and mechanical flexibility can be achieved concurrently utilizing metal-decoration method and all 2D material platforms

In this work, we report on the effect of metal-decoration on the gas-sensing properties of 2D hybrid-structures consisting of molybdenum disulfide (MoS_2_) flakes and graphene electrodes. The device showed an obviously higher response to NO_2_ gas molecules (>1.2 ppm) as well as NH_3_ (>10 ppm). It was found that metal nanoparticles (NPs) increase the selectivity for a certain gas molecule. Palladium NPs enhanced the response to NH_3_ gas molecule with respect to NO_2_. However, aluminum NPs improved NO_2_ sensitivity. The synergistic combination of metal NPs and the 2D hybrid layers could be also employed to form Pd:MoS_2_ flexible devices, showing no serious change in sensing performance even after 5000 bending cycles. Thus, metal-decorated 2D hybrid-structures could enable highly sensitive and endurable gas sensors for wearable electronic sensing platforms.

## 2. Experimental Section

### 2.1. CVD Synthesis of Graphene

A Ni/Ti film with thickness of 300/30 nm was deposited on a SiO_2_/Si wafer using an e-beam evaporator. In order to remove the oxide layer formed on the Ni film, the wafer was annealed at 300 °C under gas condition with Ar (2000 sccm)/H_2_ (80 sccm). Finally, the graphene film was synthesized at 900 °C under Ar (2000 sccm)/H_2_(80 sccm)/CH_4_ (20 sccm) for 5 min, and then the quartz tube was cooled.

### 2.2. Fabrication of Gas-Sensing Devices Consisting of MoS_2_ Channels and Graphene Electrodes

We exfoliated multilayer MoS_2_ flakes for the active channel using a conventional Scotch tape method [32] and then transferred them onto a SiO_2_/Si substrate. A multilayer graphene film for electrodes, synthesized on another Si substrate with Ni film using CVD, was patterned using conventional photolithography and reactive ion etching (RIE; O_2_, 20 sccm; pressure, 200 mTorr; and power, 50 W). After spin-coating a poly(methyl methacrylate) (PMMA, 495k, A2, MicroChem, Westborough, MA, USA) supporting layer and then etching Ni film, the patterned graphene electrode was then delivered onto the SiO_2_/Si substrate with MoS_2_ flakes. The flexible device needed an additional transfer process as follows: After spin-coating PMMA again onto the SiO_2_/Si substrate, the entire device region including MoS_2_ and graphene was transferred onto a polyimide flexible substrate by etching the SiO_2_ sacrificial layer using buffered oxide etchant. Finally, the PMMA layer was removed with acetone.

### 2.3. Metal-Decoration on MoS_2_-Based Devices

Metal NPs were deposited on transferred MoS_2_ flakes using a thermal evaporator. Specifically, Pd or Al metal with very thin thickness of 1 nm, monitored by a quartz crystal thickness monitor, was deposited on the MoS_2_ at deposition rate of ~0.3 Å/s under pressure of ~10^−6^ torr. Metal (Pd or Al) atoms nucleate on the atomically flat surface of MoS_2_ and then coalescence happens *via* a movement of the atoms, forming NPs. No post treatment was required for the formation of such NPs.

### 2.4. Gas-Sensing Measurement

Gas-sensing measurements were carried out under analyte gas (NO_2_ or NH_3_) diluted with dry air in a closed chamber. Concentrations of analyte gases were modulated by changing the flow ratio of analyte to air gas. Recovery of sensing test was done by supplying dry air and heating the device. Using a 2401 source meter (Keithley, Cleveland, OH, USA) a resistance signal of the gas-sensing devices kept monitored. An operation temperature of 150 °C was selected for optimizing the gas-sensing characteristics [24].

## 3. Results and Discussion

### 3.1. Gas-Sensing Characteristics of MoS_2_ with Graphene Electrode

**Figure 1:**
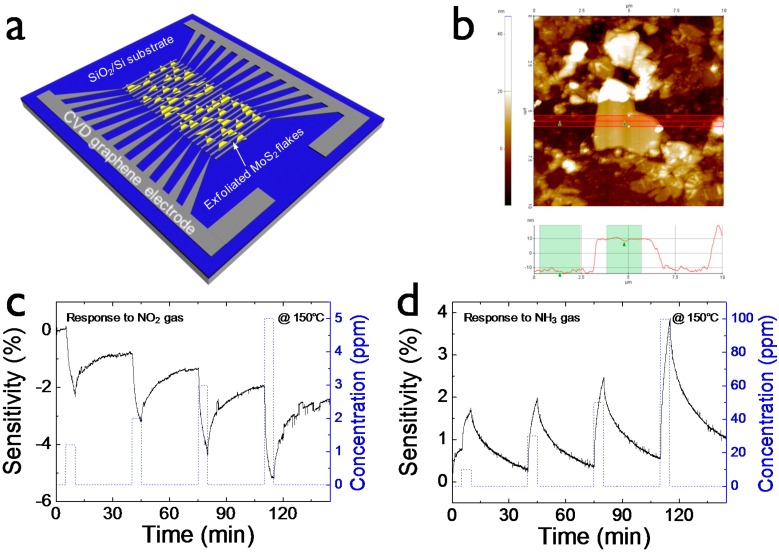
(**a**) Schematic image of the gas-sensing device consisting of mechanically exfoliated MoS_2_ flake channels and CVD-synthesized graphene electrodes; (**b**) AFM morphology of MoS_2_ flake connecting patterned graphene lines; Bottom indicates a height profile of MoS2. Transient response to (**c**) NO_2_ concentration of 1.2 to 5 ppm and (**d**) NH_3_ concentration of 10 to 100 ppm, respectively. All gas-sensing tests were carried out at an operating temperature of 150 °C.

Figure 1a shows a schematic drawing of the gas-sensing device (referred to as graphene/MoS_2_ device) comprised of mechanically exfoliated MoS_2_ flakes and CVD-synthesized graphene which function as an active channel for gas molecule detection and electrode for charge collection, respectively. Interdigitated electrode array structure was selected for higher device yield by increasing a probability that MoS_2_ flakes bridge two graphene electrode lines. The height of the MoS_2_ flakes connecting two graphene electrode lines was measured to be approximately 20 nm (Figure 1b). Flakes randomly distributed on the substrate have some thickness variation in the range of a few tens of nanometer scale.

We monitored the resistance change of the graphene/MoS_2_ device when exposed to analyte gas (NO_2_ or NH_3_). Sensitivity was defined as ∆R/R_a_ = (R_g_ − R_a_)/R_a_, where R_a_ and R_g_ indicates the resistance of the sensor under air and analyte gas, respectively. Figure 1c shows the sensitivity of the graphene/MoS_2_ sensing device under exposure to NO_2_ gas concentrations of 1.2, 2, 3, and 5 ppm. All gas-sensing tests were carried out at 150 °C operating temperature since its gas-sensing performance was optimized under this condition. Gas molecules can be strongly adsorbed onto the surface of 2D materials [33]. For desorption of the adsorbed molecules, a thermal or light energy source is necessary, enabling reversible sensing characteristics. Specifically, NO_2_ gas molecules resulted in negative sensitivity (*i.e.*, resistance decrease). With the increase of NO_2_ concentration, the absolute sensitivity value also increased. On the contrary, NH_3_ gas molecules caused positive sensitivity (*i.e.*, resistance increase), as seen in Figure 1d. As the NH_3_ concentration increased from 10 to 100 ppm, the sensitivity also increased. At the same concentration, the response to NO_2_ was much more sensitive responded than to NH_3_. It is responsible for relatively high adsorption energy of NO_2_ molecules [34,35]. The resistance change of the gas-sensing device is based on charge-transfer phenomena between gas molecules and 2D materials [26]. It is well-known that NO_2_ and NH_3_ analyte gas molecules commonly behave as electron acceptors and electron donors, respectively [5]. Interestingly, the graphene/MoS_2_ gas sensor worked as if it is a p-type device. We propose that the high work function of graphene (4.7 eV) makes it easily hole transport toward the valence band edge of MoS_2_ [36]. It is unlike the n-type behavior commonly found in low work function metal-MoS_2_ junction devices where electrons are a major carriers governing the charge transport. Thus, hole carriers involved in charge transfer of graphene/MoS_2_ device are modulated by gas molecules.

### 3.2. Metal Decoration Effect on Graphene/MoS_2_ Gas-Sensing Properties

**Figure 2:**
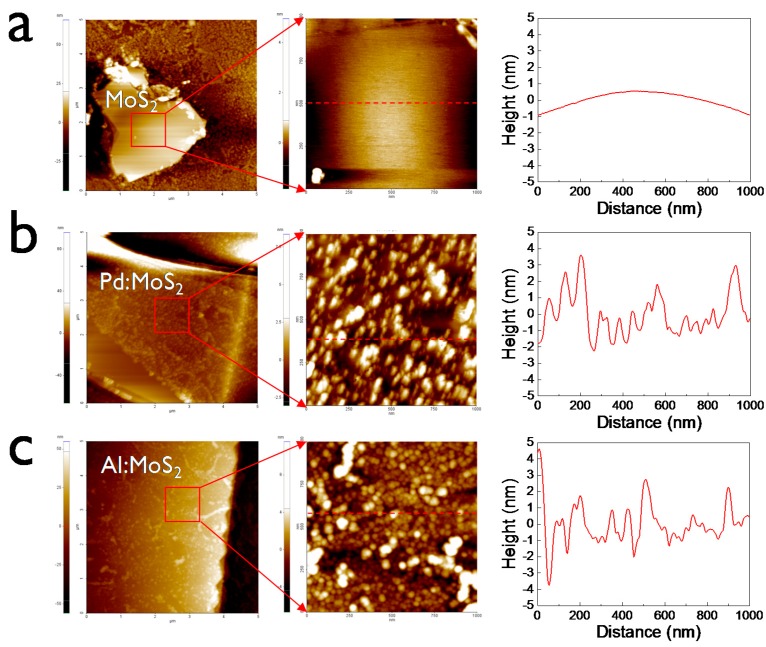
AFM morphology images and height profiles of (**a**) MoS_2_, (**b**) Pd:MoS_2_, and (**c**) Al:MoS_2_ on Si/SiO_2_ substrate.

In order to investigate the influence of metal NPs on the gas-sensing properties of MoS_2_, various metal NPs were deposited on the MoS_2_. We made three kinds of sensing devices with bare MoS_2_ and MoS_2_ decorated with Pd or Al NPs (denoted as MoS_2_, Pd:MoS_2_, and Al:MoS_2_ device, respectively). Before the electrical tests, we examined the surface morphology and height profiles of the metal-decorated MoS_2_. Basically, the morphology of MoS_2_ without metal NPs was ultra-smooth and flat (Figure 2a), indicating the layered structure of van der Waals materials.

**Figure 3:**
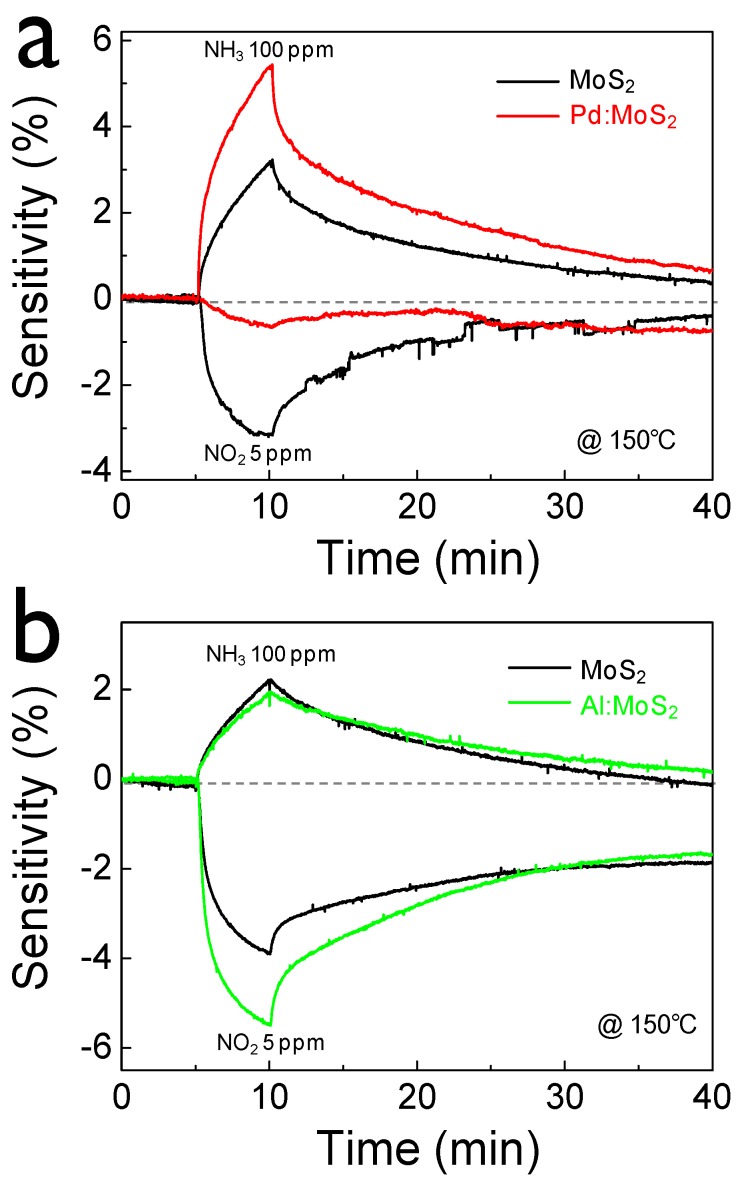

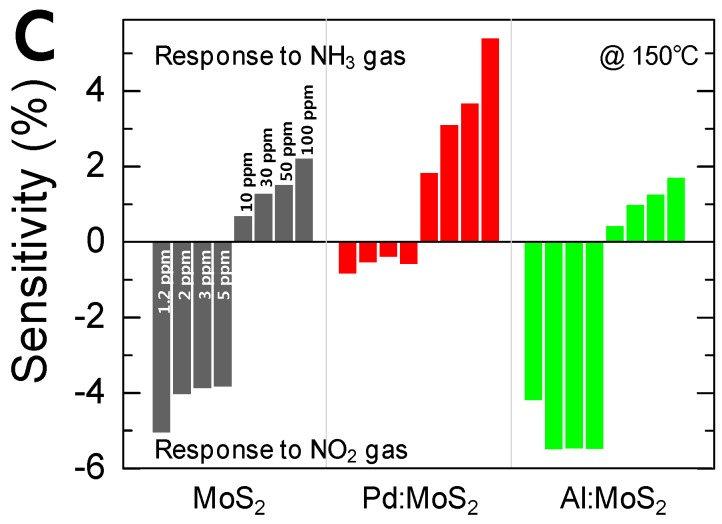
Transient gas responses under NH_3_ 100 ppm and NO_2_ 5 ppm before/after (**a**) Pd decoration on MoS_2_ and (**b**) Al decoration on MoS_2_; (**c**) Sensitivity summary of MoS_2_, Pd:MoS_2_, and Al:MoS_2_.

As shown in Figure 2b, Pd NPs were randomly distributed on the MoS_2_ surface and they aggregated in part. On the other hand, in the case of Al:MoS_2_, Al formed relatively dense and more fine particles. The morphology properties might strongly depend on physical interaction between MoS_2_ and metals. The relatively weak interaction between Al metal and MoS_2_ forms fine NPs. To investigate the effect of metal NPs on the gas-sensing properties, we checked the gas-sensing characteristics before/after the formation of metal NPs. After the deposition of Pd NPs on MoS_2_, NH_3_ sensitivity (@ 100 ppm) increased by 70% while NO_2_ sensitivity (@ 5 ppm) decreased significantly (Figure 3a). However, the opposite trend was observed for the case of Al:MoS_2_ (Figure 3b). Specifically, the NO_2_ sensitivity of the Al:MoS_2_ device was enhanced by around 40%. On the contrary, Al NPs decreased the response to NH_3_ slightly. Also, we tested the gas response characteristics under different concentrations. From the gas-sensing test results under various gas concentrations (Figure 3c), it was found that Al NPs on MoS_2_ film is most effective to detect NO_2_ gas. Pd NPs was more helpful for detecting NH_3_ gas on MoS_2_. Consequently, Pd NPs improved NH_3_ selectivity to NO_2_ while Al NPs increased the selectivity of NO_2_ to NH_3_. In particular, the sensor with a continuous metal network film shows negligible gas responses since the charge transport of the device is dominated by the metal conductive path rather than the MoS_2_ semiconducting channel.

The effects of metal decoration on sensing properties have been explained by chemical sensitization or electronic sensitization [37]. A chemical sensitization effect indicates that metal NPs on a sensing film increase the overall active sensing surface area and thereby the adsorption sites of gas molecules. Electronic sensitization can be also considered as a potential mechanism of the sensitivity tuning for each different metal [37]. Thus, it would be potentially agreeable that changes in the sensitivity in our devices are dominated by the latter model rather than the former. For more clarity, the band-bending attributed to the charge transfer between metal (Pd or Al) and semiconductor MoS_2_ was proposed (Figure S1 of Supplementary Information). Pd metal with high work function increases the hole concentration of the graphene/MoS_2_ device [38]. Such a p-doping effect of Pd on MoS_2_ enhances the gas response to NH_3_ (an electron donor molecule). This is similar to the previous study in which SnO_2_ nanocrystals act as strong p-type dopants for MoS_2_ nanosheets [39]. However, low work function Al metal depletes the hole carriers of the device and thereby increases the sensitivity of NO_2_ (an electron withdrawing molecule). Metal decoration on graphene/MoS_2_ hybrid structures modifies the electronic properties, which in turn modulates the gas-sensing characteristics. Thus, it would be a useful and simple strategy for achieving a highly selective sensor to a certain analyte gas molecule.

**Figure 4:**
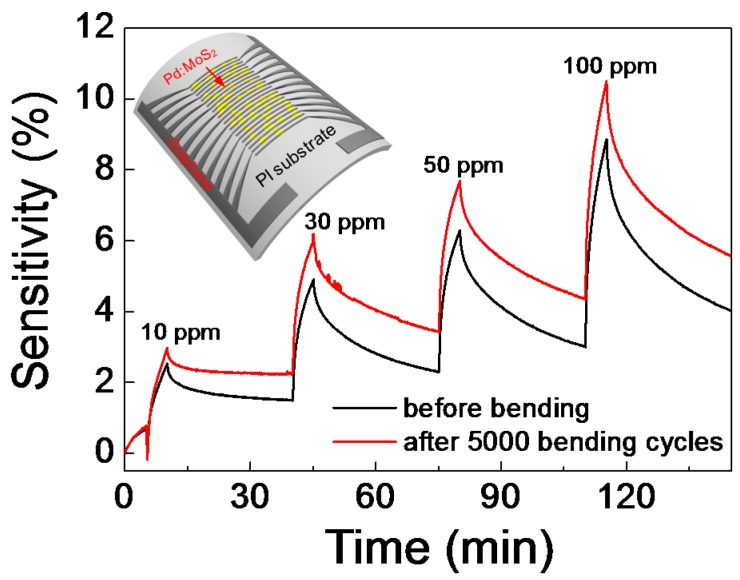
NH_3_ gas response characteristics of flexible Pd:MoS_2_ gas-sensing device before/after the bending cycle test. The inset shows a 3D schematic image of the bent device.

Next, we turned our focus to potential uses of the flexible gas sensor. The whole device region of Pd:MoS_2_ including graphene electrodes was transferred from a SiO_2_/Si wafer to a polyimide (PI) substrate (see Experimental Section for the detailed method). The NH_3_ gas-sensing characteristics of the device were obtained at 150 °C, which is necessary for reversible sensing tests (Figure 4). The glass transition temperature of the PI film is high over 250 °C so that the device can endure the heating conditions. Bent condition with a bending radius of 1.9 mm was applied to the flexibility test. The inset of Figure 4 shows a schematic image of Pd:MoS_2_ with graphene electrodes on PI substrate. Even if there is an upward shift in sensitivity with increasing gas concentration, the device did not experience any serious change in terms of sensing performance of the Pd:MoS_2_ flexible device before/after 5000 bending cycles. The cyclic gas-sensing performance was also tested for feasibility, showing a highly reproducible sensing behavior over repetitive sensing cycles (Figure S2 of Supplementary Information). Thus, highly sensitive and reliable gas-sensing performance was achieved in the flexible 2D hybrid structure with metal NPs, indicating a promising route toward the development of wearable electronic sensors.

## 4. Conclusions

Herein, we demonstrated metal-decorated gas-sensing devices based on a 2D graphene/MoS_2_ hybrid structure. Metal NPs could modulate the electronic properties of the 2D device, thereby modulating the selectivity towards a certain gas molecule. Specifically, Pd NPs electronically sensitized NH_3_ gas molecules while Al NPs enhanced NO_2_ sensitivity. The hybridization process of metal NPs and 2D materials could enable highly sensitive flexible gas sensors along with high bending endurance and stability. Ultimately, the strategy could open an avenue toward wearable sensing platforms.

## Supplementary Files

Supplementary File 1
