# Supplementary material for: Metal Decoration Effects on the Gas-Sensing Properties of 2D Hybrid-Structures on Flexible Substrates
Source: Sensors (Basel). 2015 Sep 25;15(10):24903–13. doi: 10.3390/s151024903 (PMC4634501; doi:10.3390/s151024903)
Supplement: Supplementary File 1 [file sensors-15-24903-s001.pdf]

OPEN ACCESS
sensors
ISSN 1424-8220
www.mdpi.com/journal/sensors

Supplementary Information

Metal Decoration Effects on the Gas-Sensing Properties of 2D Hybrid-Structures on Flexible Substrates. *Sensors* 2015, *15*, 24903-24913

Byungjin Cho [1,*], Jongwon Yoon [2], Sung Kwan Lim [3], Ah Ra Kim [1], Sun-Young Choi [1], Dong-Ho Kim [1], Kyu Hwan Lee [4], Byoung Hun Lee [2,3], Heung Cho Ko [2] and Myung Gwan Hahm [1,*]

[1] Advanced Functional Thin Films Department, Surface Technology Division, Korea Institute of Materials Science (KIMS), 797 Changwondaero, Sungsan-Gu, Changwon, Gyeongnam 642-831, Korea; E-Mails: kimahra86@kims.re.kr (A.R.K.); tjsdud2995@kims.re.kr (S.-Y.C.); dhkim2@kims.re.kr (D.-H.K.)

[2] School of Materials Science and Engineering, Gwangju Institute of Science and Technology (GIST), 261 Cheomdan-gwagiro, Buk-Gu, Gwangju 500-712, Korea; E-Mails: jwyoon@gist.ac.kr (J.Y.); bhl@gist.ac.kr (B.H.L.); heungcho@gist.ac.kr (H.C.K.)

[3] Department of Nanobio Materials and Electronics, Gwangju Institute of Science and Technology (GIST), 261 Cheomdan-gwagiro, Buk-Gu, Gwangju 500-712, Korea; E-Mail: lsk8410@gist.ac.kr

[4] Electrochemistry Department, Surface Technology Division, Korea Institute of Materials Science (KIMS), 797 Changwondaero, Sungsan-Gu, Changwon, Gyeongnam 642-831, Korea; E-Mail: lgh1636@kims.re.kr

* Authors to whom correspondence should be addressed; E-Mails: bjcho@kims.re.kr (B.C.); mghahm@kims.re.kr (M.G.H.); Tel.: +82-55-280-3631 (B.C.); +82-55-280-3633 (M.G.H.)

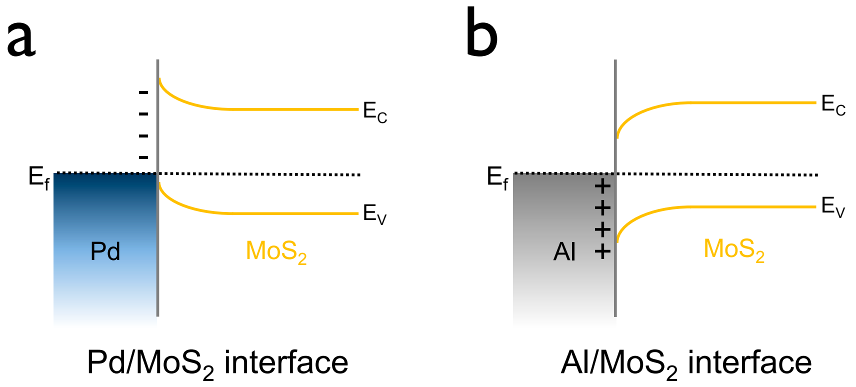

Figure S1. Diagrams of the band-bending on (**a**) Pd/MoS_2 and (**b**) Al/MoS_2 interfaces. The E_f is Fermi level of Pd and MoS_2 aligned after junction formation. The E_C and E_V are the conductance and valance band edges of MoS_2, respectively.

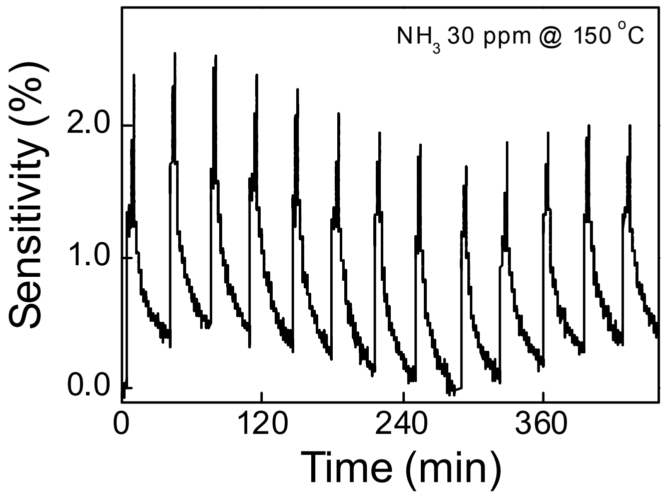

Figure S2. Cyclic gas-sensing performance of flexible $Pd:MoS_2$ gas-sensing device under NH_3 30 ppm, showing a highly reproducible sensing behavior.

© 2015 by the authors; licensee MDPI, Basel, Switzerland. This article is an open access article distributed under the terms and conditions of the Creative Commons Attribution license (http://creativecommons.org/licenses/by/4.0/).
